# Spectral features of nuclear DNA in human sperm assessed by Raman Microspectroscopy: Effects of UV-irradiation and hydration

**DOI:** 10.1371/journal.pone.0207786

**Published:** 2018-11-20

**Authors:** Raul Da Costa, Sandra Amaral, Klaus Redmann, Sabine Kliesch, Stefan Schlatt

**Affiliations:** 1 Centre for Reproductive Medicine and Andrology, University of Münster, Münster, Germany; 2 Biology of Reproduction and Stem Cell Group, Center for Neuroscience and Cell Biology (CNC), University of Coimbra, Coimbra, Portugal; 3 Institute for Interdisciplinary Research, University of Coimbra, Coimbra, Portugal; National Cancer Institute, UNITED STATES

## Abstract

Raman Microspectroscopy represents an innovative tool for the assessment of sperm biochemical features otherwise undetectable by routine semen analysis. Previously, it was shown that induced DNA damage can be detected in smeared sperm by this technique. This novel readout may be of value for clinical settings especially if it can be transferred to living cells. Yet, starting with living sperms this study was carried-out using a variety of conditions to disclose the Raman features of sperm nuclei under different hydration conditions and UV exposure. Human sperm were immobilized and Raman spectra were obtained from individual sperm as repeated measurements. To create conditions with controlled DNA damage, sperm samples were exposed to ultraviolet light. Several media were used to evaluate their effect on Raman spectra in aqueous conditions. To substantiate differences between the experimental conditions, the spectra were analyzed by Principal Component Analysis. We observed that spectra of sperm nuclei obtained in different solutions showed a qualitatively unchanged spectral pattern showing the principal signals related to DNA. Evaluating the effect of ultraviolet light generated the finding that spectra representing DNA damage were only observed in dry conditions but not in aqueous medium. Thus, Raman microspectroscopy was successfully applied for sperm analysis in different conditions, among them in live spermatozoa in aqueous solution during the initial measurement, revealing the principle use of this technique. However, implementation of Raman spectroscopy as a technique for clinical sperm analysis and selection may be especially relevant when DNA evaluation can be established using live sperm.

## Introduction

In the last decade many new strategies were explored to update and amend the well-established routine procedures for semen analysis recommended by WHO [1]. It represents the primary component of male fertility assessment which provides information on cellular composition of the ejaculated sample and some functional aspects of spermatozoa. However, its prognostic and diagnostic value for testicular function and the general state of the male genital tract and health of the patient is limited [1–3]. This fact is especially evident in cases where natural conception does not occur, despite sperm parameters being within “normal reference” values. These cases are classified as “idiopathic infertility” and include nearly 30% of the men referred to fertility treatments [4,5]. This scenario is far from ideal and highlights the immediate need for novel and more informative assays in semen analysis.

Evenson et al. [6] described a flow cytometric procedure (sperm chromatin structure assay, SCSA) for assessment of sperm nuclear DNA integrity. DNA Fragmentation index (DFI) was defined as a quantitative readout for sperm analysis. The usefulness of this parameter in experimental settings has been established and its clinical application for prediction of the success of fertility treatments is under evaluation and discussion. It is evident that high levels of sperm DNA damage determined by SCSA are associated with detrimental effects on natural and assisted conception [7,8]. Sperm DNA damage has been related to several features such as impaired fertilization, poor embryo development, low implantation rates, increased rates of spontaneous abortion and decreased live-birth rates [7–11]. The implementation of sperm DNA integrity assays therefore represents a decisive step towards modern and functional seminal analysis, having the potential to be a decisive factor for the diagnosis and prognosis in several clinical scenarios [12].

However, although numerous methods are available for the analysis of sperm DNA, all of them are either invasive or destructive and those sperm used for analysis cannot be used for fertilization following diagnostic procedures [1,13]. An innovative tool to potentially avoid such drawbacks is Raman Microspectroscopy (RM) [14]. This technique uses only intrinsic properties of light, whose intensity can be modulated and no labeling or extraction procedures are needed. It has therefore the capability to fingerprint the chemical composition of a biological sample without compromising the structural and functional integrity of the cells [15–17]. Additionally, thanks to its coupling to confocal microscopes, RM can be performed with very high lateral spatial resolution and minimal depth of field (i.e., below 1 μm), allowing the analysis of individual cells and organelles. These attributes render this technique ideal for the innocuous assessment of sperm biochemical features that otherwise would remain undetectable by routine methods [1,16,17].

The application of RM for the study of the biochemical characteristics of sperm is not new. Almost 30 years ago, Kubasek et al. [18] evaluated DNA extracted from salmon sperm to determine its conformational state. However, after this initial study there was a gap on Raman research applied to sperm and it was only in the last decade that the potential of the technique to identify chemical signatures underlying morphology and mitochondrial status of human sperm was recognized [19,20]. In recent studies carried out in our laboratory, it was shown that experimentally induced sperm DNA damage could be disclosed in smeared samples through the detection of a band located at ~1050 cm^-1^ in the Raman spectrum [21]. This peak and its ratio to a peak at ~1094 cm^-1^ correspond to the symmetric stretching vibration of the DNA phosphate backbone and can therefore be considered as a readout of DNA biochemical changes. It correlates with the DFI scores obtained by SCSA [13]. Moreover, the presence of this band was consistently reported regardless the source of damage [13,21,22]. Our findings were confirmed by other authors [23]. This technique has also been applied for the non-invasive sex assessment in bovine semen, being able to distinguish X and Y bearing sperm [24,25]. Recently our group reported that several organelle-specific (sperm head, midpiece and tail) spectral features are present in human, monkey, mouse and sea urchin sperm, which could be considered potential spectral markers for sperm quality [26].

Another highly exciting avenue might be offered if the evaluation of semen samples could be performed on live sperm in aqueous medium [27]. RM might be advantageous over other vibrational spectroscopy techniques, such as infrared spectroscopy as in principle it might be independent of the hydration status. As yet all studies on were carried out using individual air-dried sperm on smears which are not suitable for clinical applications or subsequent physiological studies. Therefore, despite promising initial findings, the non-destructive nature of the technique has not been truly exploited. Thus, the aim of this work was to explore the application of RM starting from living sperm in aqueous conditions to test different media and to evaluate Raman spectra from un-damaged and UV-damaged sperm nuclei under different hydration status.

## Materials and methods

### Samples

Human normozoospermic (in accordance to World Health Organization [1]) semen samples from six (n = 6) donors were collected by masturbation after 2–5 days of sexual abstinence and prepared by swim-up. A written informed consent was obtained, and the project was approved by the ethics committee of the University Clinic of Münster.

### Experimental design

#### Effect of different solutions/media

To test the effect of media on sperm, three solutions (saline, Phosphate-Buffered Saline (PBS) and Human Tubal Fluid (HTF)) differing substantially in chemical composition (S1 Table) were selected. After swim-up spermatozoa were transferred to these conditions. Sperm were then immobilized using Suprasil slides (Quartzglas Heinrich QGH, Aachen, Germany) coated overnight with poly-lysine (1mg/ml). In subsequent experiments HTF media was used if not otherwise noted.

#### Effect of hydration conditions on the DNA spectrum of sperm exposed to ultraviolet light

As we described previously [28], sperm damage was induced experimentally by exposure of the immobilized sperm in HTF media to 30 min of ultraviolet light (UVB 312 nm). UVB-induced sperm DNA damage was confirmed by SCSA (S1 Fig).

### Confocal Raman microspectroscopy

Raman spectra of sperm nuclei were obtained as established by Sánchez et al. [13]. Briefly, to acquire single spectra, the cells were focused and the laser was positioned in the postacrosomal region of the sperm head where the highest concentration of DNA is located [21], in that position one single-point scans (two accumulations of 20 sec each) were performed, the acquisitions were done at 600 grooves/mm diffraction grating with slit and pinhole apertures of 100 μm (pixel resolution of 2.2 cm^-1^/CCD pixel and spectral resolution of 6.7 cm^–1^) within a wavelength range of 600 to 1,800 cm^-1^, to pointing at the sperm a 632.8 nm He-Ne laser (~15 mW) was used with an Olympus BX41 microscope (x100 objective, NA = 0.9 at 210 μm working distance), which resulted in a laser spot size of 0.85 μm and a radiation power of ~2200 kW/cm^2^. This radiation power was tested in our previous studies and it was shown not to induce photodamage [21], The optics was coupled to a confocal Raman system (LabRAM Aramis, HORIBA Jobin Yvon S.A.S, Lille, France). Z profile mapping was conducted for each acquisition to obtain optimal signal to noise ratio. Labspec 6 software (6.3.40.3; HORIBA Jobin Yvon S.A.S., Lille, France) was used for all the acquisitions. Daily, before spectra acquisition, the machine was calibrated using a silicon reference slide and laser position was verified.

For the spectral acquisition of the experiments concerning UV exposure and hydration status, fifty independent sperm (n = 50) from a “sperm population” were randomly selected for each condition and fifty particular (n = 50) “individual sperm” that were followed through the time in all condition using a coordinate system (S2 Fig); briefly, an “X” shaped scratch was made on the surface of the slide to set it as a reference point. Next, the sperm surrounding that point were evaluated and after each of the different treatments and conditions (e.g. UV exposure), the scratch on the slide was located to search and assess the same individual spermatozoa that were previously interrogated. The population-based analysis was intended to validate and confirm our findings using cohorts of spermatozoa As mentioned six (n = 6) different ejaculates were used, five for testing hydration conditions on the DNA spectrum of sperm exposed to ultraviolet light (2 for sperm population and 3 for individual sperm) and another to test the different solutions/media, for which a new set of 10 sperm was used for each medium.

### Spectral analysis

In order to increase spectral quality, the background signals (S3 Fig) were subtracted and the spectra were baseline corrected, smoothed, normalized by unit vector and set to zero, using LABSpec. The mean spectrum of each treatment was obtained and peak assignment was done having as reference the works of Talari et al. [29] and Amaral *et al*. [26] which we highly recommend for further details about the spectral profile of sperm cells. The measurements of all individual spectra (over the 650–1,800 cm-1 spectral range) were analyzed by Standard principal component analysis (PCA) and plotted using the R 3.4.1 and RStudio 1.0.153 [30]. PCA analysis is a dimension reduction analysis that permits to better visualize the variation present in high dimensional data allowing in this way the identification of patterns within them and highlighting their similarities and differences [21,31,32]. Briefly, the data corresponding to each experiment were grouped together, and the corresponding PC vectors were computed. Next, the scores with respect to each sperm group were computed, and the scores with respect to PC vector one and two were plotted against each other, in different colors for each single group to visualize clusters and the separation of data sets, reflecting the differences between the compared groups and allowing to derive information regarding the basis for discrimination when the loadings corresponding to each PC are compared to the Raman spectra.

## Results

### Effect of different solutions/media

**Fig 1:**
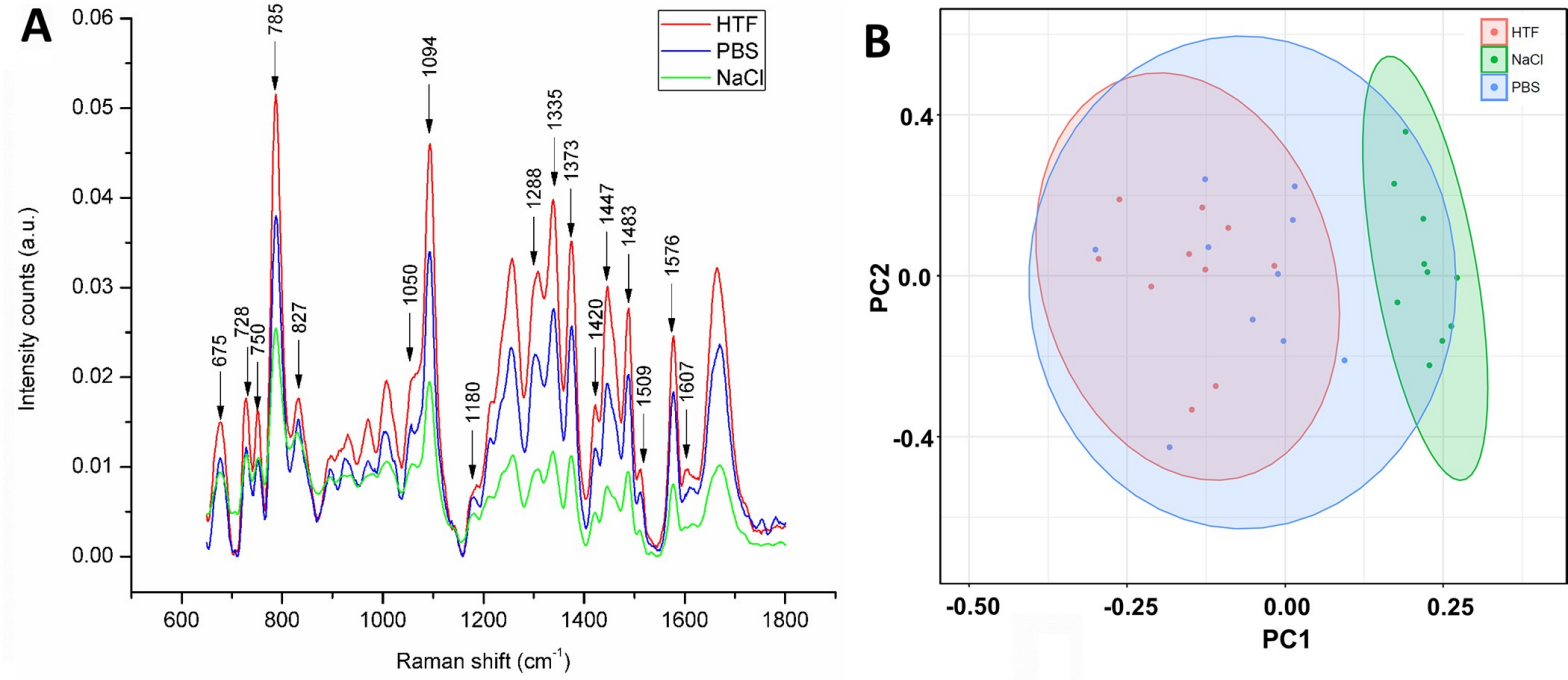
Effect of different solutions/media. **(**A) Average Raman spectrum (n = 10) of the sperm DNA in different solutions/media in which the most common peaks mentioned in the text are pointed out. (B) Principal component analysis of individual spectra. The confidence interval of the ellipses is 95% considering a normal distribution.

The effect of different solutions on the spectral characteristics of nuclear DNA of human sperm can be observed in Fig 1A, where the qualitative aspects of the pattern remains widely unchanged in each of the three conditions and all the main peaks associated with DNA are observed in all three conditions (Fig 1A and Table 1). Equally, it is evident that the intensity of the average spectra increases according to the complexity of the solution. The bands corresponding to the DNA spectrum evaluated in HTF medium have the highest intensities followed by PBS and saline.

**Table 1:** Main bands corresponding to the sperm DNA Raman spectrum and their assignments according to Amaral et al. [26].

**RS**^a^ **(cm-1)**	**Assignation**
**~675**	- G ring breathing mode
**~728**	- A (ring breathing mode of DNA/RNA bases)
**~750**	- DNA, tryptophan
**~785**	- U, T, C (ring breathing modes in the DNA/RNA bases). DNA backbone O–P–O
**~1094**	-Symmetric PO2− stretching vibration of the DNA backbone; C-N of proteins
**~1109**	-DNA/RNA backbone
**~1180**	A,C,G,T; Tyrosine; Phenylalanine
**~1288**	-A, T, amide III (proteins), = CH (lipids)
**~1335**	- A, G (ring breathing modes in the DNA bases)
**~1373**	-T, A, G; Tryptophan, porphyrins and lipids; C-H (proteins)
**~1420**	-A, G (ring breathing modes of the DNA/RNA bases)
**~1447**	-CH_2_ bending mode of proteins and lipids (1446, 1447)
**~1483**	-G and A (ring breathing modes and purine bases)
**~1509**	- C, A (ring breathing modes in the DNA bases)
**~1576**	-G, A ring breathing modes; -8-Oxoguanosine
**~1607**	-C = C phenylalanine, tyrosine
**~1660**	-C = C (lipids); Amide I (proteins);

^a^ Raman shift

**Fig 2:**
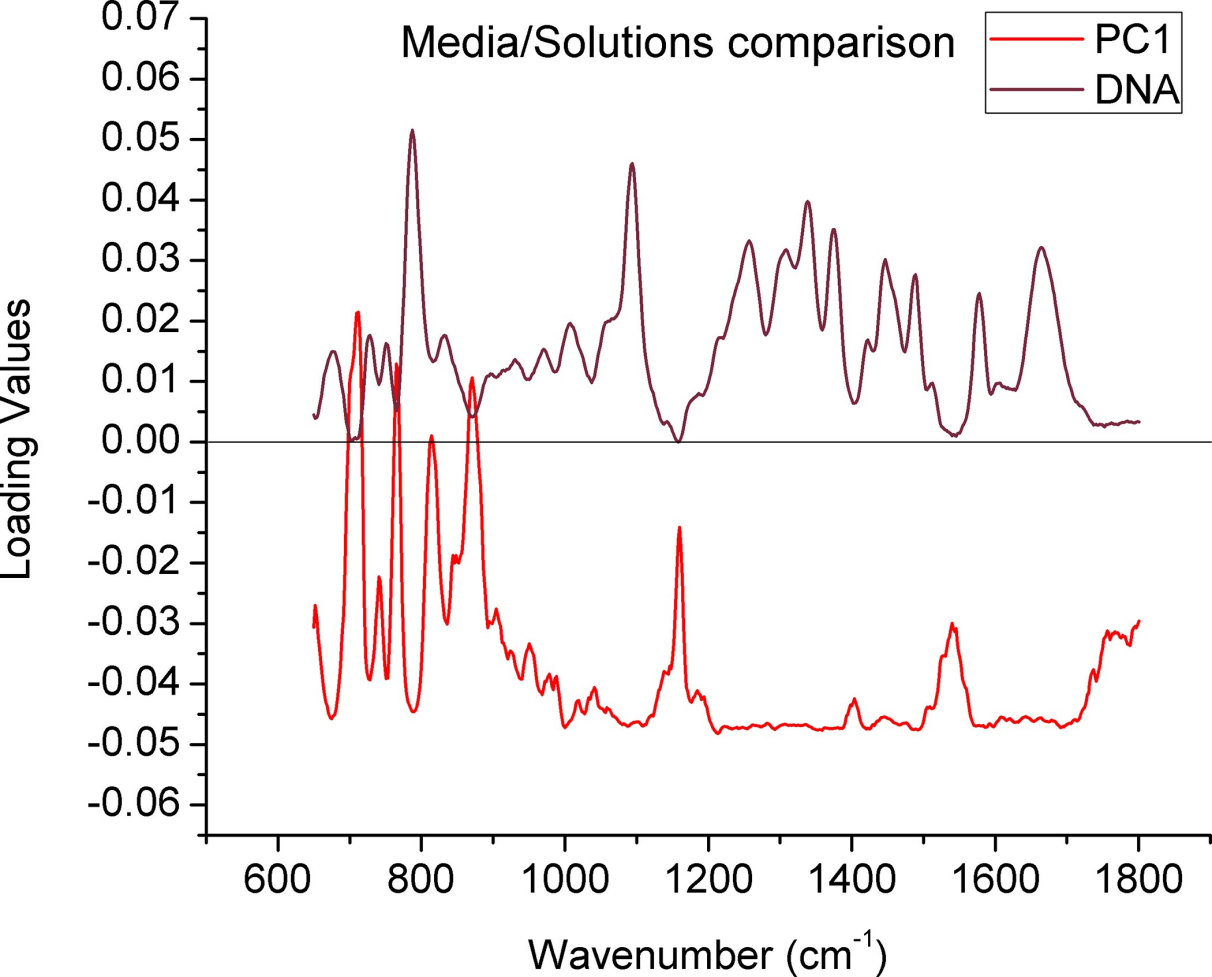
Comparison between the loading values of the PC1 in Fig 1 and a typical Raman spectrum of the sperm DNA. The values were generated from the comparison of the different media/solutions. The black line denotes zero in the loading values.

PCA provides further insight into the source of the spectral variability and could confirm if this differences are significant, in this sense, even though some variances can be observed in the dots distribution of the PCA score plotting (Fig 1B) where the sperm evaluated in saline appear to form a relatively independent group, this distinction provided by the PC1, it does not seem to relay on a relationship between the loading values in PC1 (Fig 2) with the spectral bands of DNA or the background signal (S3 Fig). Further analysis of scores related to subsequent PCs (PC3 and PC4) (S4 Fig) do not show any distinction between the groups. Based on these data, in subsequent experiments HTF given that is the most commonly used medium in assisted reproduction procedures [33] was used for spectral acquisition.

### Effect of hydration conditions on the DNA spectrum of sperm exposed to ultraviolet light

**Fig 3:**
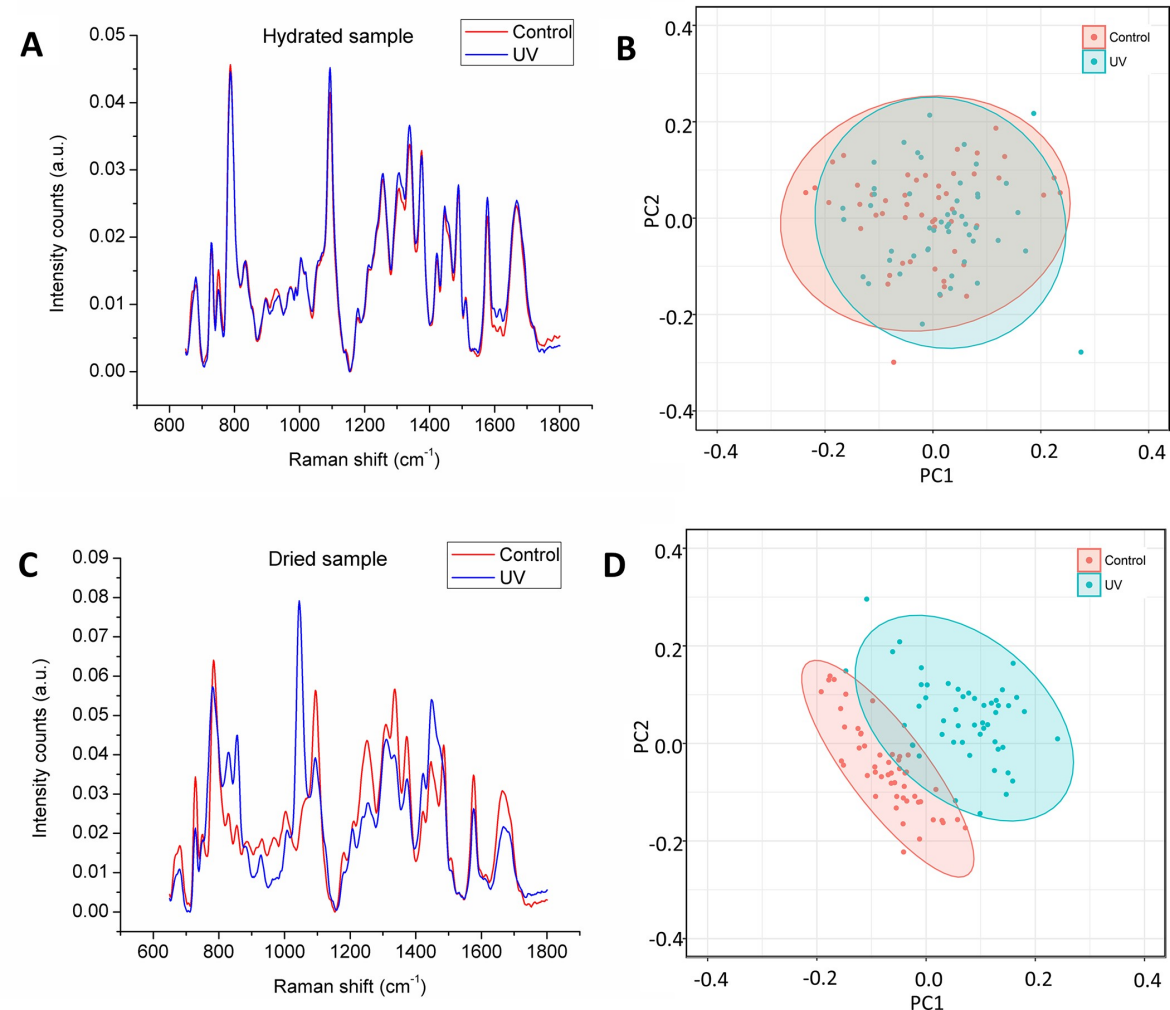
Effect of hydration conditions on the DNA spectrum of individual sperm exposed to ultraviolet light (UV). The results shown in this figure correspond to particular sperm cells that were followed through the time in all condition using a coordinate system. (A) Average Raman spectrum (n = 50) of the DNA of non-exposed (control) and UV light exposed (UV) sperm in hydrated conditions. (B) Principal component analysis of single spectra of sperm in hydrated conditions. (C) Average Raman spectrum (n = 50) of the DNA of non-exposed (control) and UV light exposed (UV) sperm in dehydrated conditions. (D) Principal component analysis of single spectra of sperm in dehydrated conditions. The confidence interval of the ellipses is 95% considering a normal distribution.

The effect of hydration on the Raman spectrum can be seen in Fig 3. When evaluating the effect of UVB-induced damage on individual immobilized sperm under hydrated conditions in HTF medium (Fig 3A), the DNA spectrum of control and UVB-treated sperm show similar patterns except for a few intensity changes in some specific bands, namely, ~750 corresponding to T ring breathing mode in nucleic acids; ~935 a protein assignment related to the C-C stretching mode of the amino acids proline and valine; ~1308 assigned to G and the A ring breathing mode, ~1335 related to the ring breathing modes in the DNA bases, specifically A and G; and ~1607 corresponding to C = C of phenylalanine and tyrosine. All these bands, despite presenting slight differences in their amplitude are clearly represented in both conditions. In this way, no previously described spectral trait of UV induced-damage is visible, this findings apply for both, individual sperm (Fig 3A) and population (S5 Fig), despite the fact that the samples exposed to UV light showed high scores of DFI when analyzed through SCSA (S1 Fig).

**Fig 4:**
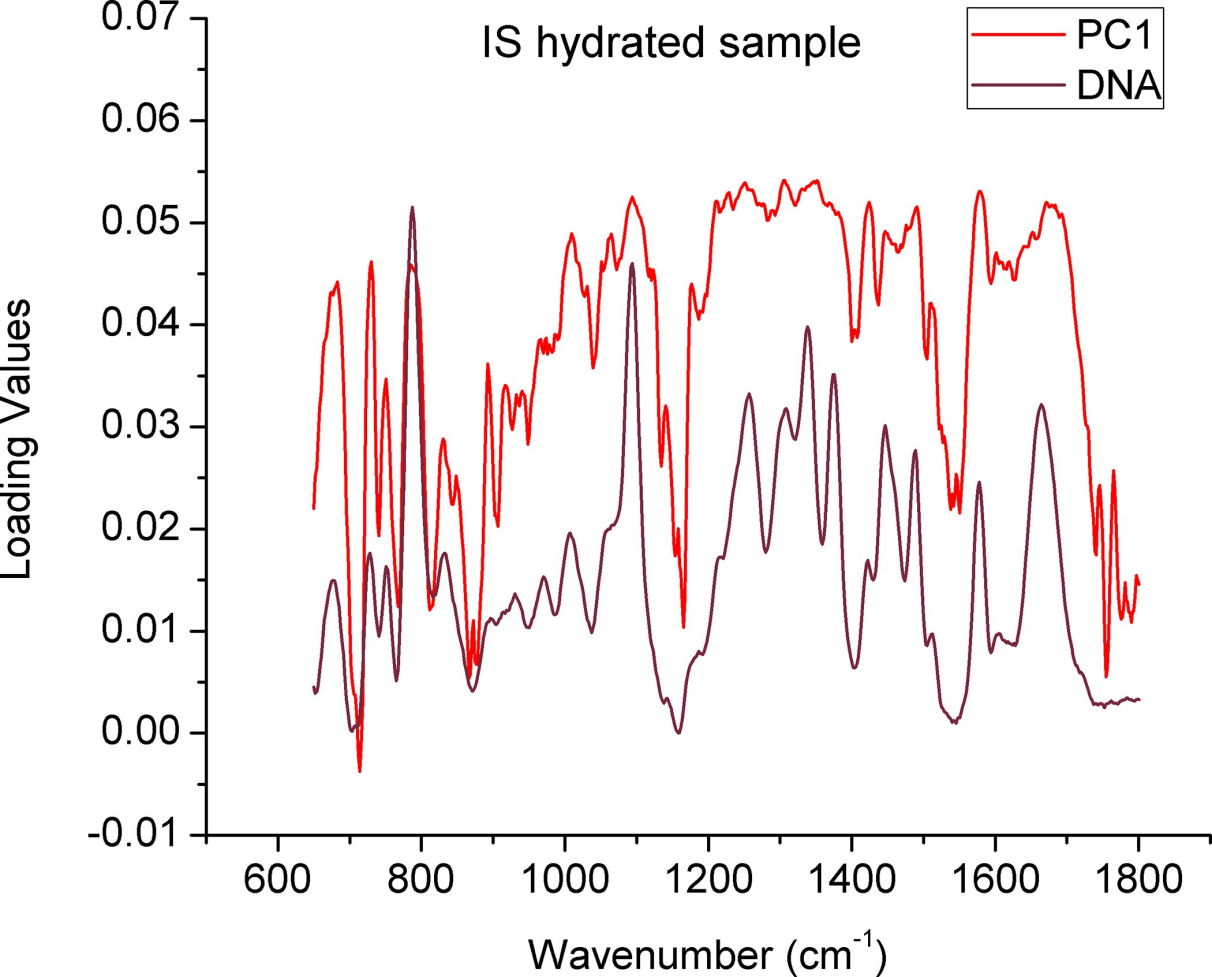
Comparison between the loading values of the PC1 in Fig 3B and a typical Raman spectrum of the sperm DNA. The values were generated from the comparison of the spectra of individual sperm (control/UV exposed) evaluated in aqueous medium.

This similarity is confirmed in the PCA plotting, where indistinguishable groups are shown (Fig 3B and S5 Fig), in the same way when observing the respective loadings values corresponding to the main PC1 (Fig 4), although a detailed analysis is complex, it can be noted that these are dominated by the spectral characteristics of the sperm DNA, which validates the fact that no distinction can be made between the groups by biochemical reasons, this applies to both individual sperm (Fig 4) and population (S6 Fig). Further analysis of scores related to subsequent PCs (PC3 and PC4) (S7 and S8 Figs) also do not show any distinction between the groups.

On the other hand, when the same individual sperm (Fig 3C) and sperm population (S5 Fig) were analyzed on air-dried smears; is possible to appreciate a drastic transformation of the sperm DNA spectrum which evidences the biochemical changes induced by UV radiation, this is obvious in the bands corresponding to nucleic acids, which is the case of the peak at ~1094 cm^-1^ which corresponds to the symmetric stretching vibration of the PO^2−^ backbone of the DNA and the band at ~1050 cm^-1^ indicative of DNA damage, which show the most dramatic changes after UV exposure in dried samples as reported by Sánchez et al. [13]. In the same way, changes are also shown in the bands at ~827 cm^-1^ representing the O–P–O stretch in DNA/RNA; the band at ~1253, ~1335 and ~1483 cm^-1^ related to the ring breathing modes of the DNA bases. Additionally, other alterations in the spectrum of other biomolecules are also visible, specifically those at ~850 cm^-1^ assigned to single-bond stretching vibrations for the amino acids proline, hydroxyproline, tyrosine and valine; and also polysaccharides; and the one at ~1446 cm^-1^, related to proteins and the methylene deformation mode from endogenous lipids also reported to be shifted by Sánchez et al. [13].

**Fig 5:**
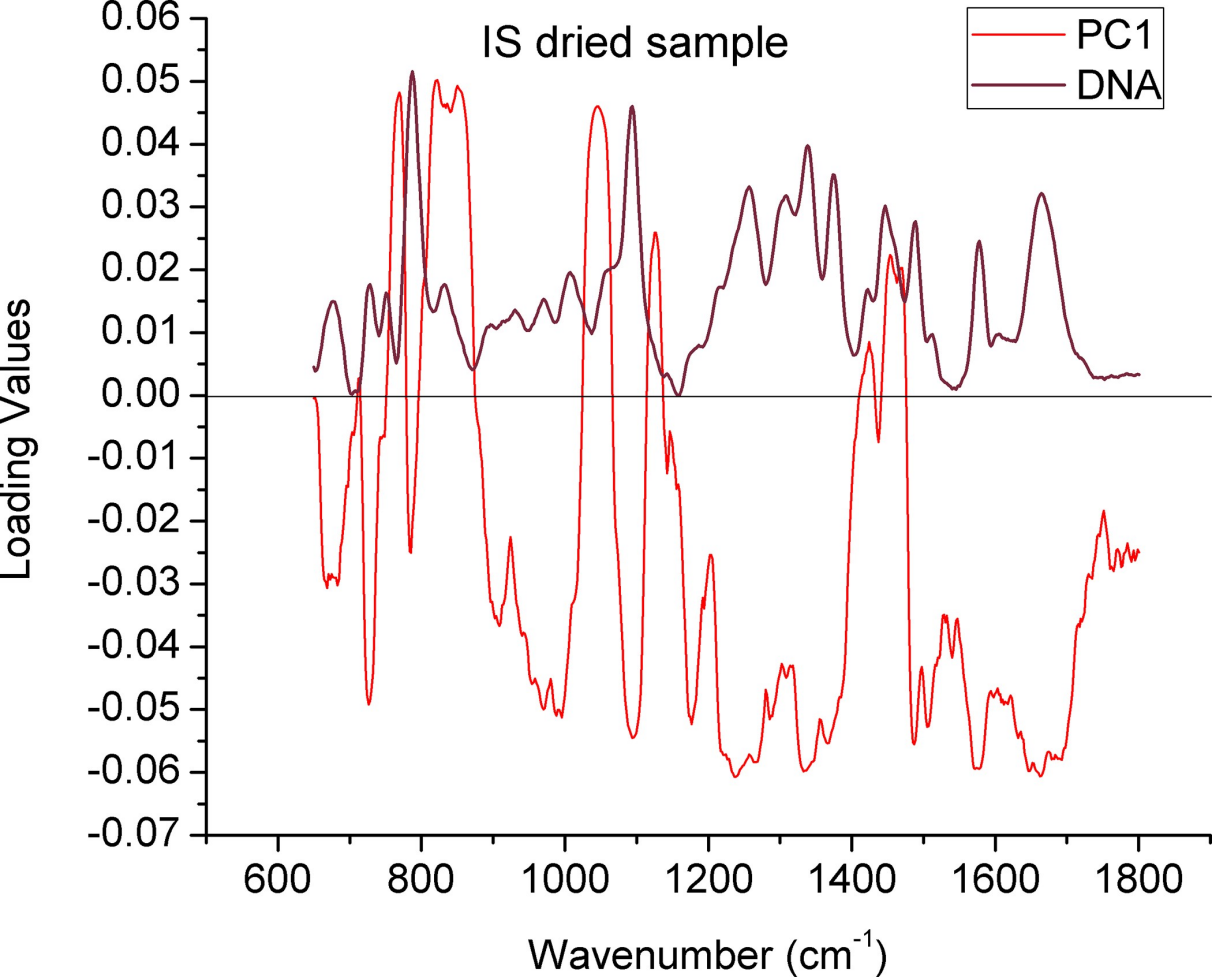
Comparison between the loading values of the PC1 in Fig 3D and a typical Raman spectrum of the sperm DNA. The values were generated from the comparison of the spectra of individual sperm (control/UV exposed) evaluated in dry conditions.

Changes are also evident in the PCA plots where the dots corresponding to control spermatozoa form a compact group followed by a more spread population representing the cells treated with UV light, both populations are located in a particular region of the plot so the area related to DNA damage can be easily recognized (Fig 3D and S5 Fig). This distinction lays partly in PC1 (Fig 5 and S9 Fig), whose loading values are comparable to the DNA spectrum of the sperm analyzed and confirms that the distinction is valid and is being made based on the biochemical characteristics of the DNA of both groups. The PCA performed with the subsequent PCs (PC3 and PC4) (S10 and S11 Figs) do not provide additional information.

## Discussion

In addition to its non-invasive and non-destructive nature, one of the most exciting features of RM is that the cell composition can be easily scanned and visualized in physiological conditions, allowing to obtain information on the main chemical moieties associated with biomolecules, without any interference of the water signal [27]. Here we evaluated if the chemical components in solutions exert an effect on the Raman spectrum of nuclear sperm. In this regard, although the intensity in some bands varies, no further qualitative changes are clearly detectable, either in the average spectra (Fig 1A) or in the PCA analysis (Fig 1B), in which although there seems to be a distinction in the group of spermatozoa evaluated in saline solution, this distinction seems to be the product of baseline oscillations and not of biochemical factors as can be seen in Fig 2, where the loading values of the PC1 seems to have no relation with the spectral characteristics of the sample. Moreover, no difference between the groups can be observed when a PCA using other PCs (3 and 4) is applied S4 Fig.

Regarding spectral intensities, it is well known that this parameter can be influenced by the ionic state of the molecules and therefore media in which the samples are embedded may have effects [34]. The efficiency of spontaneous Raman scattering is very low [16,27]; therefore, to record conventional Raman spectra, analytical concentrations greater than 0.01M are required when a typical total Raman scattering cross-section of ca.10−29 cm^2^ per molecule is taking in consideration [35]. Most of the components in solution are therefore undetectable, explaining why the spectral pattern of the DNA is not modified by the composition of the medium.

Qualitatively, all bands previously reported by our group [27], namely ~1094, ~1180, ~1373, ~1420, ~1489, ~1509 and ~1576 cm^-1^ are visible in the spectra of sperm DNA, indicating that the clear visualization of the signals in aqueous solution, is in principle possible given that the composition of the medium does not exerts a mayor effect on the characteristics of the sperm DNA spectrum when determined by RM.

Regarding the effect of hydration conditions on the DNA spectrum of sperm exposed to ultraviolet light, no effect of exposure to UV light could be detected in the sperm spectrum evaluated in aqueous medium (Fig 3A), these observations are supported by the PCA (Fig 3B) where the groups evaluated are indistinguishable, in this case, unlike that in the comparison of the media, the loading values of PC1, do show reciprocity with the spectral characteristics of the samples (Fig 4), which confirms that the biochemical differences whose presence was assessed through SCSA (S1 Fig), are not detectable by Raman microspectroscopy in aqueous conditions.

Contrariwise, in dry conditions the differences between the average spectra when comparing control sperm and sperm exposed to UV light are clearly visible (Fig 3C), showing that in the same cells that were evaluated in aqueous conditions, the latent biochemical changes that in those conditions they were not perceptible, are now easy to asses. These differences in this populations can also be observed in the PCA plotting (Fig 3D), where the distinction is based essentially in PC1, whose loading values are comparable to the DNA spectrum of the analyzed cells (Fig 5 and S9 Fig), which confirms that the distinction is being made based on the biochemical characteristics of DNA in both groups. In this way, when observing the loading values that make up PC1 (Fig 5), it can be observed that the distinction between control and UV-exposed spermatozoa is given by the negative values in the case of the control, which correspond to the peaks at ~675, ~730, ~785, ~1094, ~1483, ~1509 and ~1579 cm^-1^. The positive values are related to the group treated with UV, which correspond to the peaks at ~827, ~1050, ~1420 and ~ 1447 cm^-1^, of which some of them have been previously associated with damage in the sperm DNA, as mentioned above. Similar observations can be found in the measurements corresponding to the sperm population (S5 and S9 Figs).

On the other hand, the explanation for the absence of spectral features indicating DNA damage after UV-irradiation is, as yet, not easy. Pascolo et al. [36] using FTIR spectromicroscopy on sperm samples exposed to oxidative stress mediated by Fenton’s reaction, reported that the DNA damage interpretation is partially compromised. Unexpected cell surface iron-oxygen precipitates were generated during Fenton’s reaction and generated a broad band which partially occupies the DNA phosphate stretching bands. However, we dismiss the presence of this compound since the DNA damage in our study was induced by UV light. In another study it was induced by exposure to an uropathogenic *E*. *Coli* [22]. We see identical outcomes irrespective of the induction of DNA damage.

On the other hand, it is known that presence of ions and water are essential in determining the conformation of a nucleic acid [37]. For instance, by Fourier-transform infrared spectroscopy (FTIR) it was proven that dehydration induces the transition of DNA from the natural B- to the A-helical conformation [36]. However, a conformational change in the DNA molecule is most likely not the reason explaining our observations. In our case, all spectra irrespective of hydration status, show a clear band at ~675 cm^-1^, which corresponds to the vibration mode of the guanine ring restated to a sugar-phosphate backbone in B-form conformation [19,38]. Furthermore, we have also detected the presence of a band at ~827 cm^-1^, in both hydration states, especially in the spectra related to UV light exposure, which has been reported as a B form marker band [39–41]. Given the detection of the B form, this seems not to be the case in our study.

In regard to the above mentioned, in spermatozoa about 85–90% of the DNA is packaged by protamines coiling the DNA into a highly dense crystalline-like structure [42]; therefore unlike in other cells its natural B-conformation may be stabilized regardless of the hydration status. It is also worth mentioning that the spectrum of pure DNA damaged by UV radiation in aqueous solution has been previously described. Specifically Ke et al. [41] proved that the irradiation of pure herring sperm DNA for 30 min or more with UV light, in aqueous solution, severely affected the DNA structure through the denaturation of purine and pyrimidine bases. Furthermore, they have shown that in aqueous medium the band at ~1095 cm^−1^ remained unchanged in the base composition/structure [41], similar to our observations.

Moreover, in the past the 1055 cm-1 peak was already addressed to solid DNA/RNA; thus, Dukor and Griffiths [43] using calf thymus DNA have shown that in the solid, the most significant difference between the two nucleic acids is the ratio of intensity of the bands corresponding to the symmetric phosphate stretching vibrations, around 1055 cm^-1^ leading to the conclution that these spectra are highly dependent on the state of hydration.

Although the exact physical-chemical nature of such dehydration-dependent changes is currently unknown to us, the effect of hydration may change when the integrity of the backbone is absent, in this sense, we hypothesize that the non-hydrated state might expose some alterations that might not be “accessible” in the hydrated state, similar to the SCSA, in which the acid-induced DNA denaturation *in situ* is a prerequisite to detect the otherwise latent damage in sperm DNA [6]. More experimental studies are needed to obtain a precise mechanistic explanation why the hydration status changes the ability to detect DNA changes in sperm DNA.

## Conclusions

In this work significant progress has been made in the development of RM applied to sperm. Raman spectra of the sperm nucleus were repeatedly obtained under different conditions starting from living cells and showing that the main spectral pattern of the sperm nuclear DNA remains unchanged regardless of the medium in which it is analyzed. However, we also found that in aqueous environment the differences between the spectrum of UVB-treated sperm and the untreated control sperm are latent, indicating an interference of the hydration status for determination of DNA integrity by Raman microspectroscopy.

## Supporting information

S1 Fig Sperm chromatin structure assay (SCSA) of sperm suspensions. (Black) Non UV-Treated sperm suspension (Control) showing a low DNA Fragmentation Index (DFI); (Blue) UV-Treated sperm suspension showing a high DFI.
(TIF)

S2 Fig Sperm immobilization and tracking for Raman analysis. (A) Composite image of 5x5 fields at 10x, where the scratch in the form of “X” which was used as a reference to follow the individual sperm is shown. (B) Zoom-in of the internal box shown in S2A composed of 12x12 images at 100x. The Labspec 6 software allows the assignation of coordinates (x,y), to which it is possible to return to analyze the same point. The red dot in B was set up as coordinate (0,0).
(TIF)

S3 Fig Signals of the background of the experiments carried out in aqueous conditions. (TIF)

S4 Fig Principal component analysis (PC3 vs PC4) of the experiments carried out in aqueous conditions to test the effect of different media/solutions in the sperm DNA spectrum. (TIF)

S5 Fig Effect of hydration conditions on the DNA spectrum of a sperm population exposed to ultraviolet light (UV). The results shown in this figure correspond to independent sperm cells that were randomly selected in each condition.(A) Average Raman spectrum (n = 50) of the DNA of non-exposed (control) and UV light exposed (UV) sperm in hydrated conditions. (B) Principal component analysis of single spectra of sperm in hydrated conditions. (C) Average Raman spectrum (n = 50) of the DNA of non-exposed (control) and UV light exposed (UV) sperm in dehydrated conditions. (D) Principal component analysis of single spectra of sperm in dehydrated conditions. The confidence interval of the ellipses is 95% considering a normal distribution.
(TIF)

S6 Fig Comparison between the loading values of the PC1 in S5B Fig and a typical Raman spectrum of the sperm DNA. The values were generated from the comparison of the spectra of sperm populations (control/UV exposed) evaluated in aqueous medium.
(TIF)

S7 Fig Principal component analysis (PC3 vs PC4) of the experiments carried out in individual sperm (control/UV exposed) evaluated in aqueous medium. (TIF)

S8 Fig Principal component analysis (PC3 vs PC4) of the experiments carried out in sperm population (control/UV exposed) evaluated in aqueous medium. (TIF)

S9 Fig Comparison between the loading values of the PC1 in S5D Fig and a typical Raman spectrum of the sperm DNA. The values were generated from the comparison of the spectra of sperm populations (control/UV exposed) evaluated in dried conditions.
(TIF)

S10 Fig Principal component analysis (PC3 vs PC4) of the experiments carried out in individual sperm (control/UV exposed) evaluated in dried conditions. (TIF)

S11 Fig Principal component analysis (PC3 vs PC4) of the experiments carried out in sperm populations (control/UV exposed) evaluated in dried conditions. (TIF)

S1 Table Solutions/media composition. (DOCX)

S1 File PC_Loadings_Fig 1B. Complete set of the PC loadings associated to the Principal Component Analysis concerning the evaluation of the effect of different media /solutions in the Raman spectrum of the human sperm DNA (Fig 1B).
(CSV)

S2 File PC_Loadings_Fig 3B. Complete set of the PC loadings associated to the Principal Component Analysis concerning the comparison of the spectra of individual sperm (control/UV exposed) evaluated in aqueous medium (Fig 3B).
(XLSX)

S3 File PC_Loadings_Fig 3D. Complete set of the PC loadings associated to the Principal Component Analysis concerning the comparison of the spectra of individual sperm (control/UV exposed) evaluated in dried conditions (Fig 3D).
(XLSX)

S4 File PC_Loadings_S5B Fig. Complete set of the PC loadings associated to the Principal Component Analysis concerning the comparison of the spectra of sperm populations (control/UV exposed) evaluated in aqueous medium (S5B Fig).
(XLSX)

S5 File PC_Loadings_S5D Fig. Complete set of the PC loadings associated to the Principal Component Analysis concerning the comparison of the spectra of sperm populations (control/UV exposed) evaluated in dried conditions (S5D Fig).
(XLSX)
