# Supplementary figures and images for: Spectral features of nuclear DNA in human sperm assessed by Raman Microspectroscopy: Effects of UV-irradiation and hydration
Source: PLoS One. 2018 Nov 20;13(11):e0207786. doi: 10.1371/journal.pone.0207786 (PMC6245842; doi:10.1371/journal.pone.0207786)

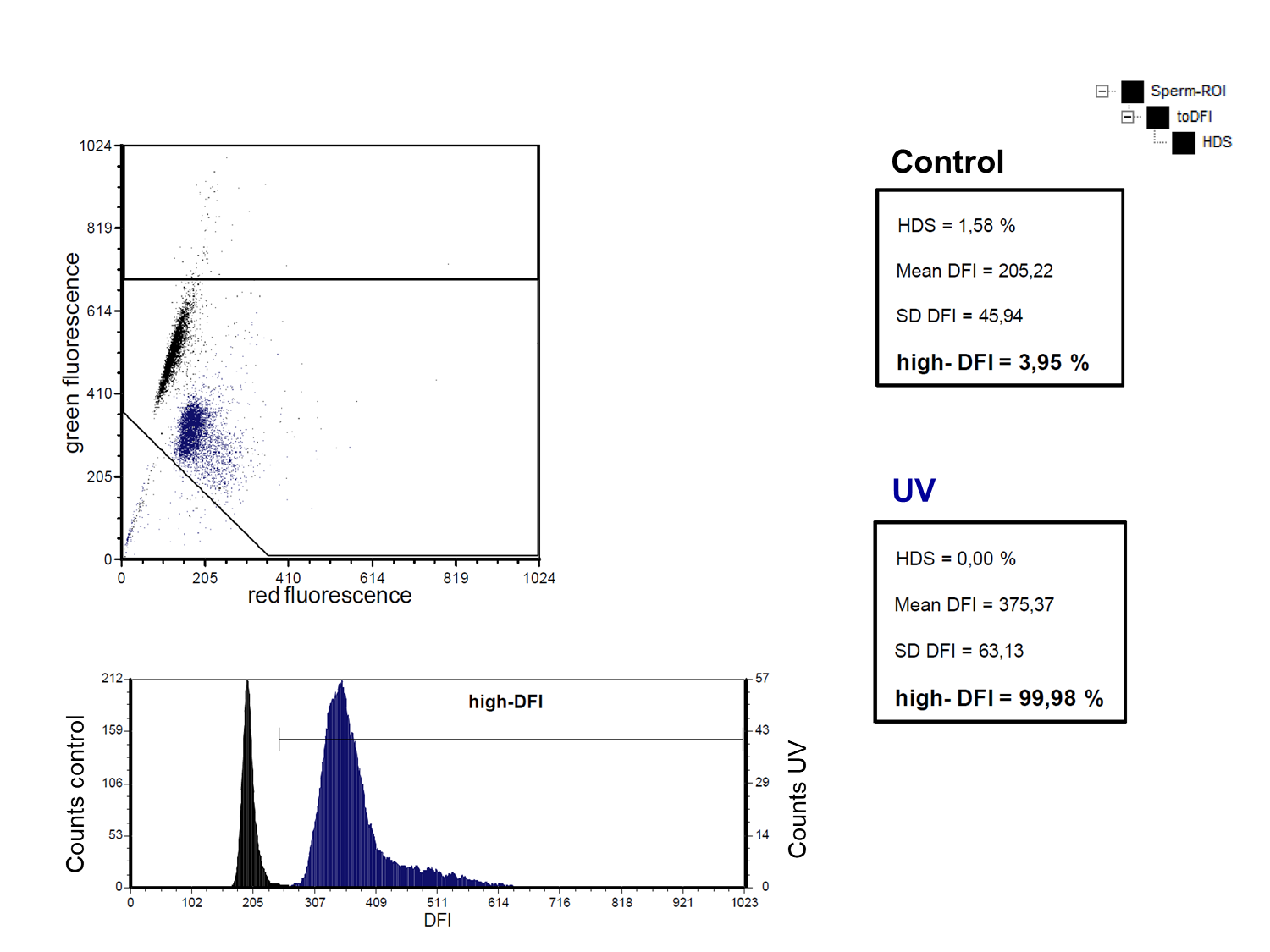

Supplement: S1 Fig — (Black) Non UV-Treated sperm suspension (Control) showing a low DNA Fragmentation Index (DFI); (Blue) UV-Treated sperm suspension showing a high DFI. (TIF) [file pone.0207786.s001.tif]

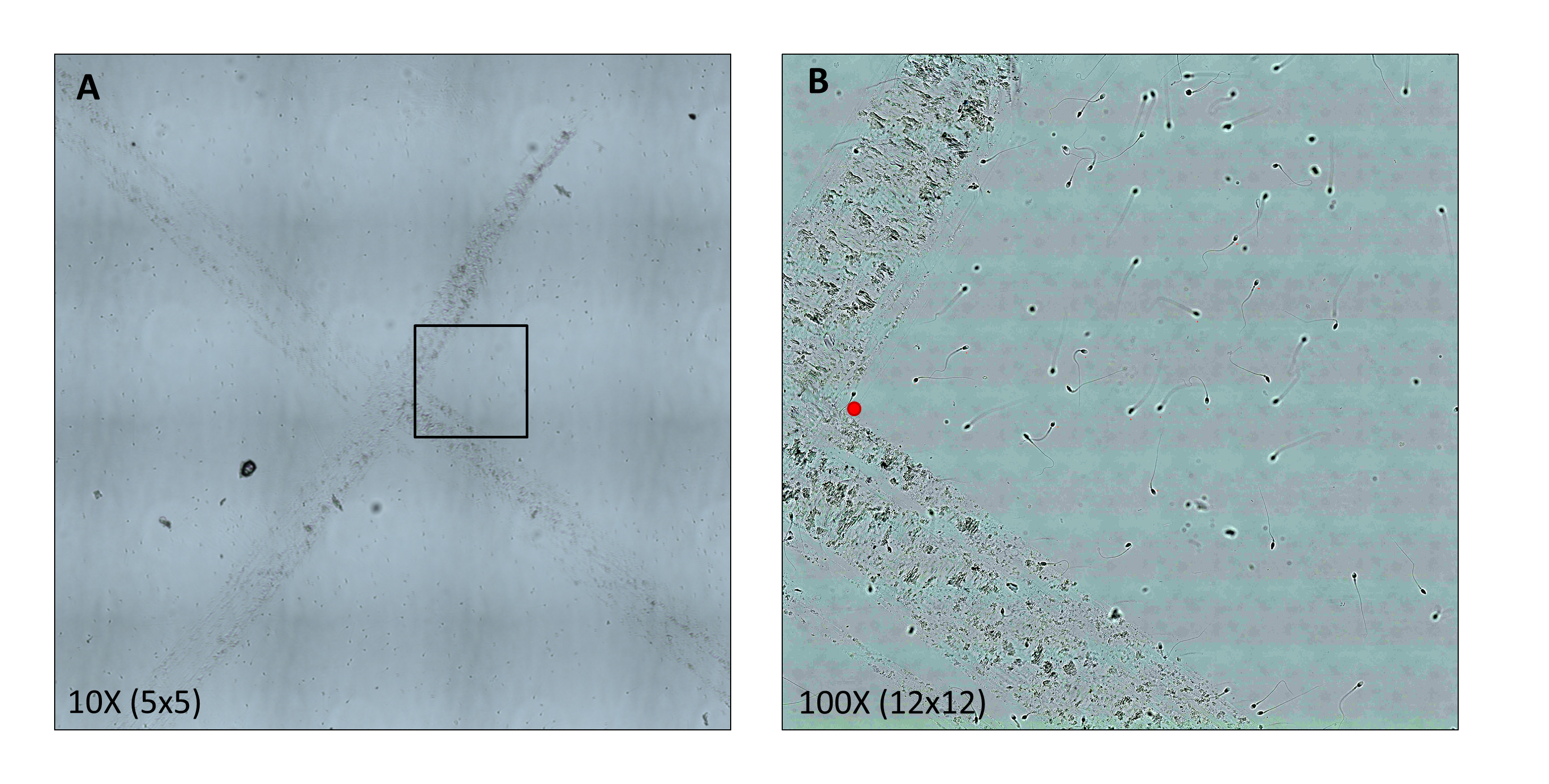

Supplement: S2 Fig — (A) Composite image of 5x5 fields at 10x, where the scratch in the form of “X” which was used as a reference to follow the individual sperm is shown. (B) Zoom-in of the internal box shown in S2A composed of 12x12 images at 100x. The Labspec 6 software allows the assignation of coordinates (x,y), to which it is possible to return to analyze the same point. The red dot in B was set up as coordinate (0,0). (TIF) [file pone.0207786.s002.tif]

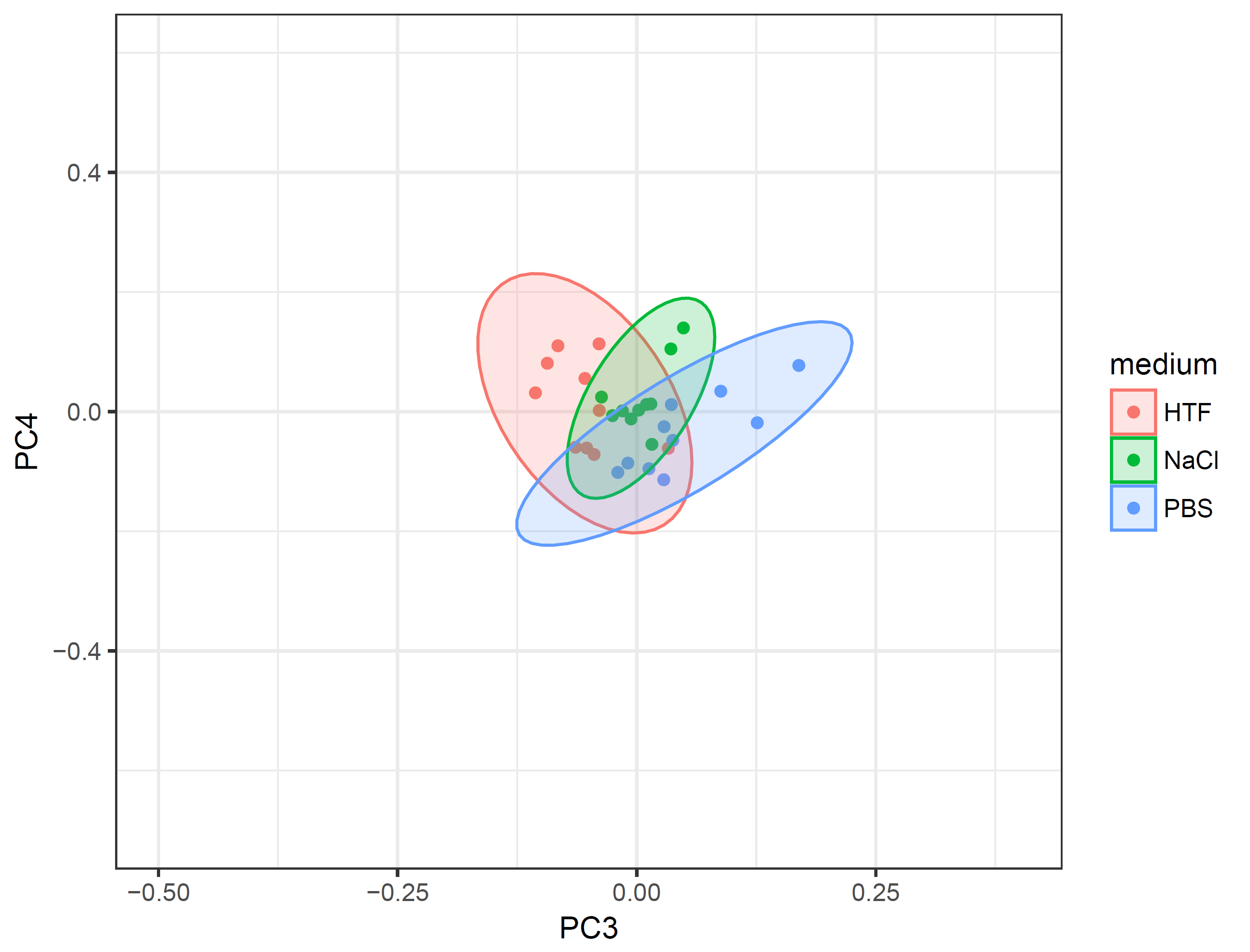

Supplement: S4 Fig — (TIF) [file pone.0207786.s004.tif]

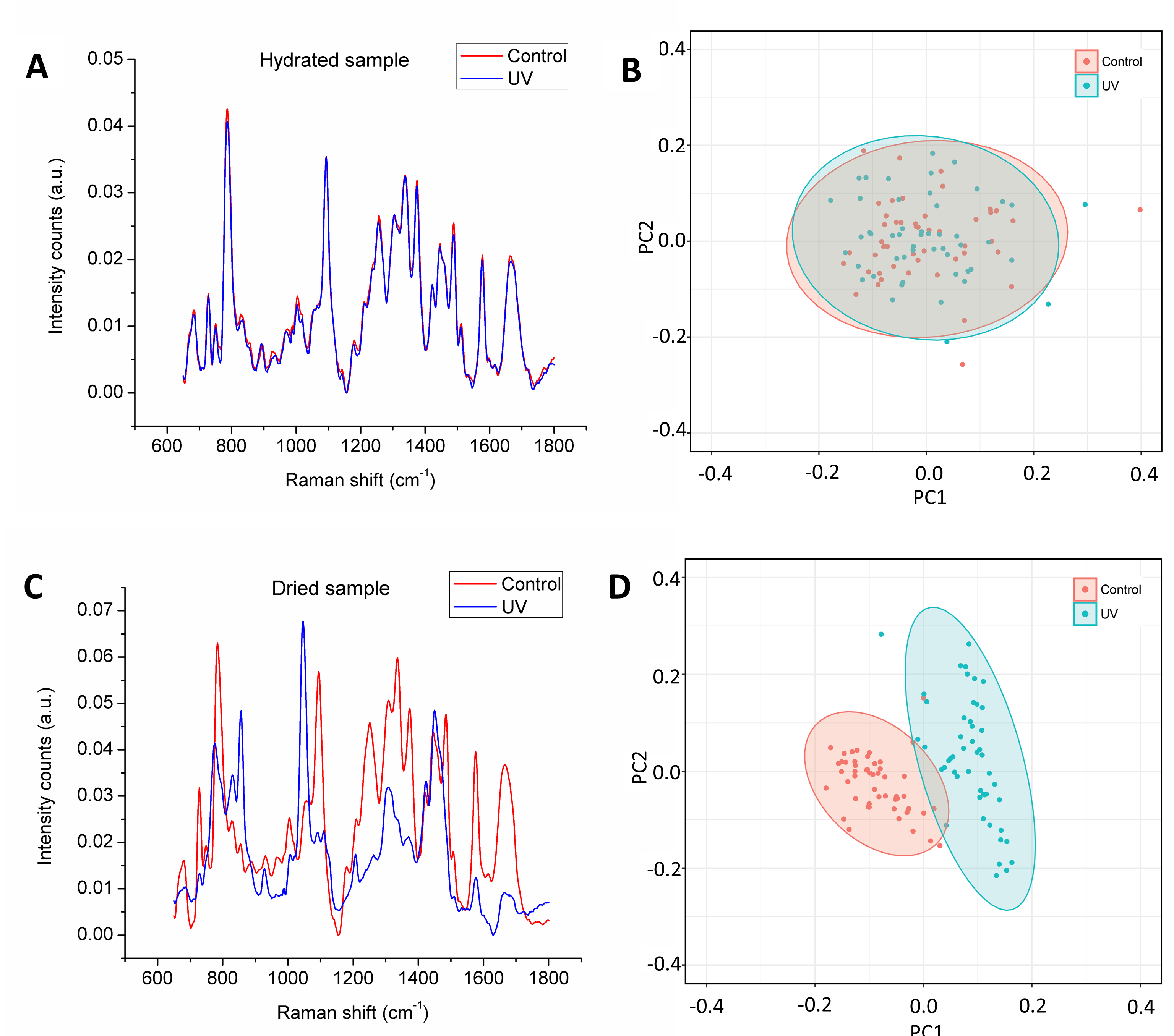

Supplement: S5 Fig — The results shown in this figure correspond to independent sperm cells that were randomly selected in each condition.(A) Average Raman spectrum (n = 50) of the DNA of non-exposed (control) and UV light exposed (UV) sperm in hydrated conditions. (B) Principal component analysis of single spectra of sperm in hydrated conditions. (C) Average Raman spectrum (n = 50) of the DNA of non-exposed (control) and UV light exposed (UV) sperm in dehydrated conditions. (D) Principal component analysis of single spectra of sperm in dehydrated conditions. The confidence interval of the ellipses is 95% considering a normal distribution. (TIF) [file pone.0207786.s005.tif]

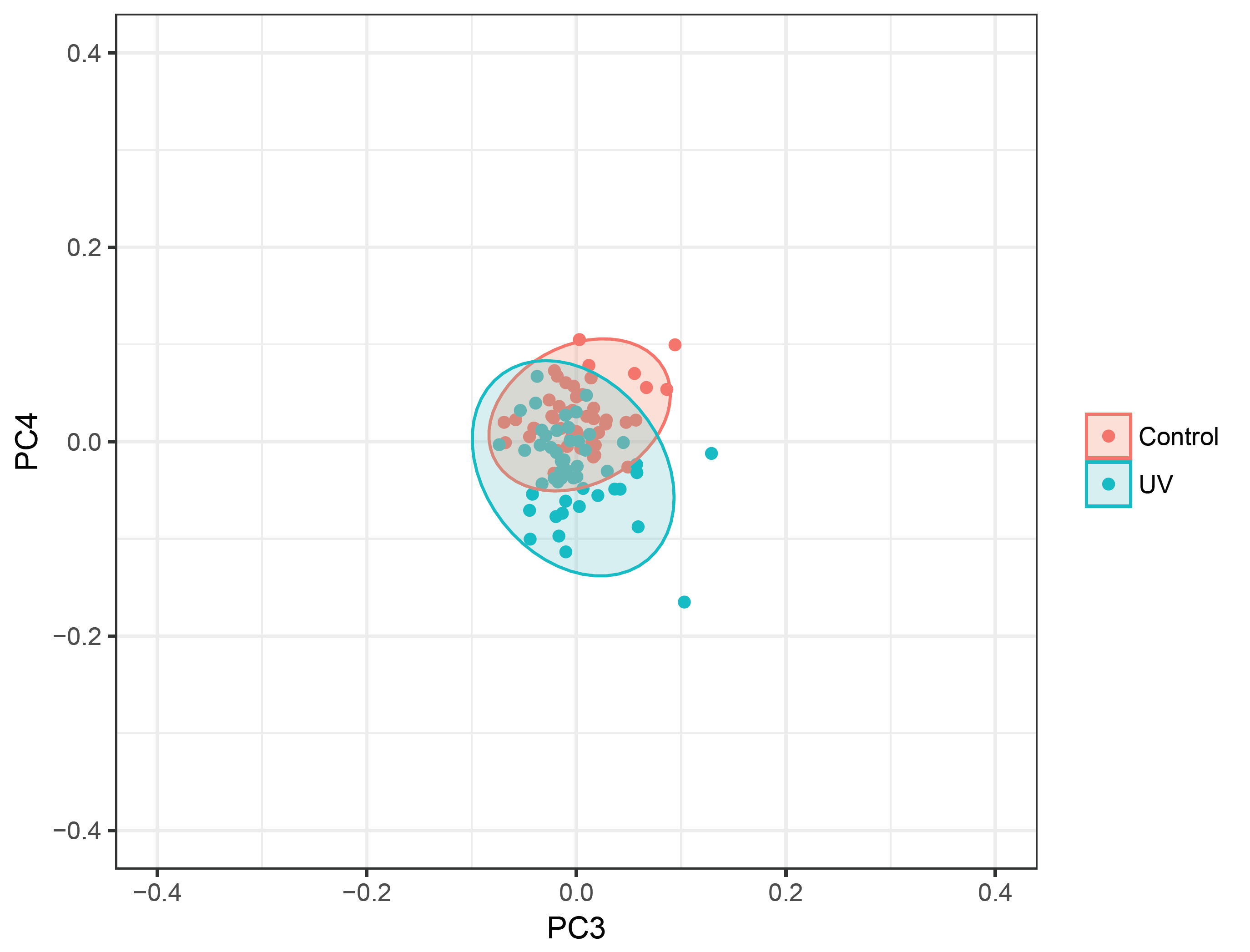

Supplement: S7 Fig — (TIF) [file pone.0207786.s007.tif]

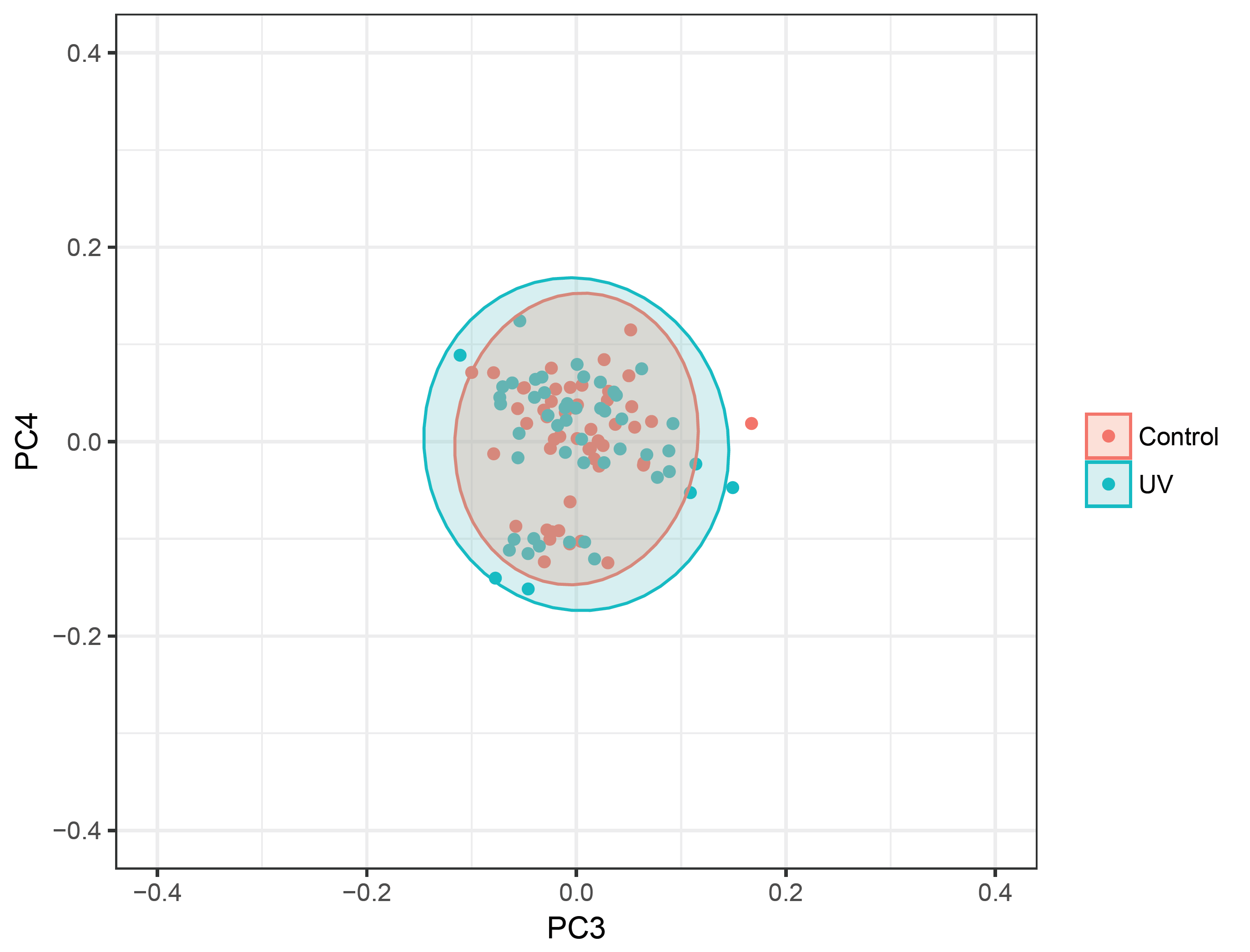

Supplement: S8 Fig — (TIF) [file pone.0207786.s008.tif]

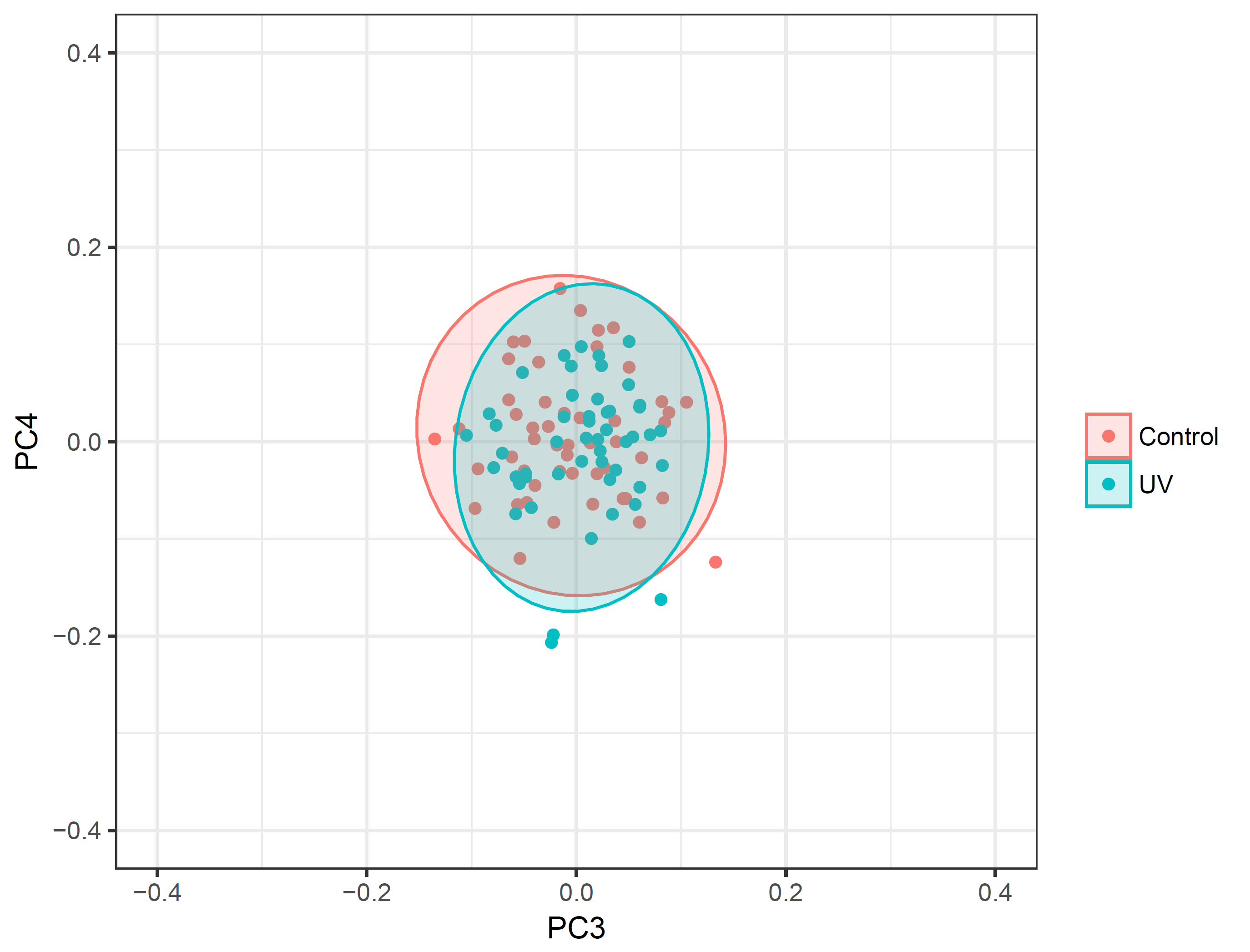

Supplement: S10 Fig — (TIF) [file pone.0207786.s010.tif]

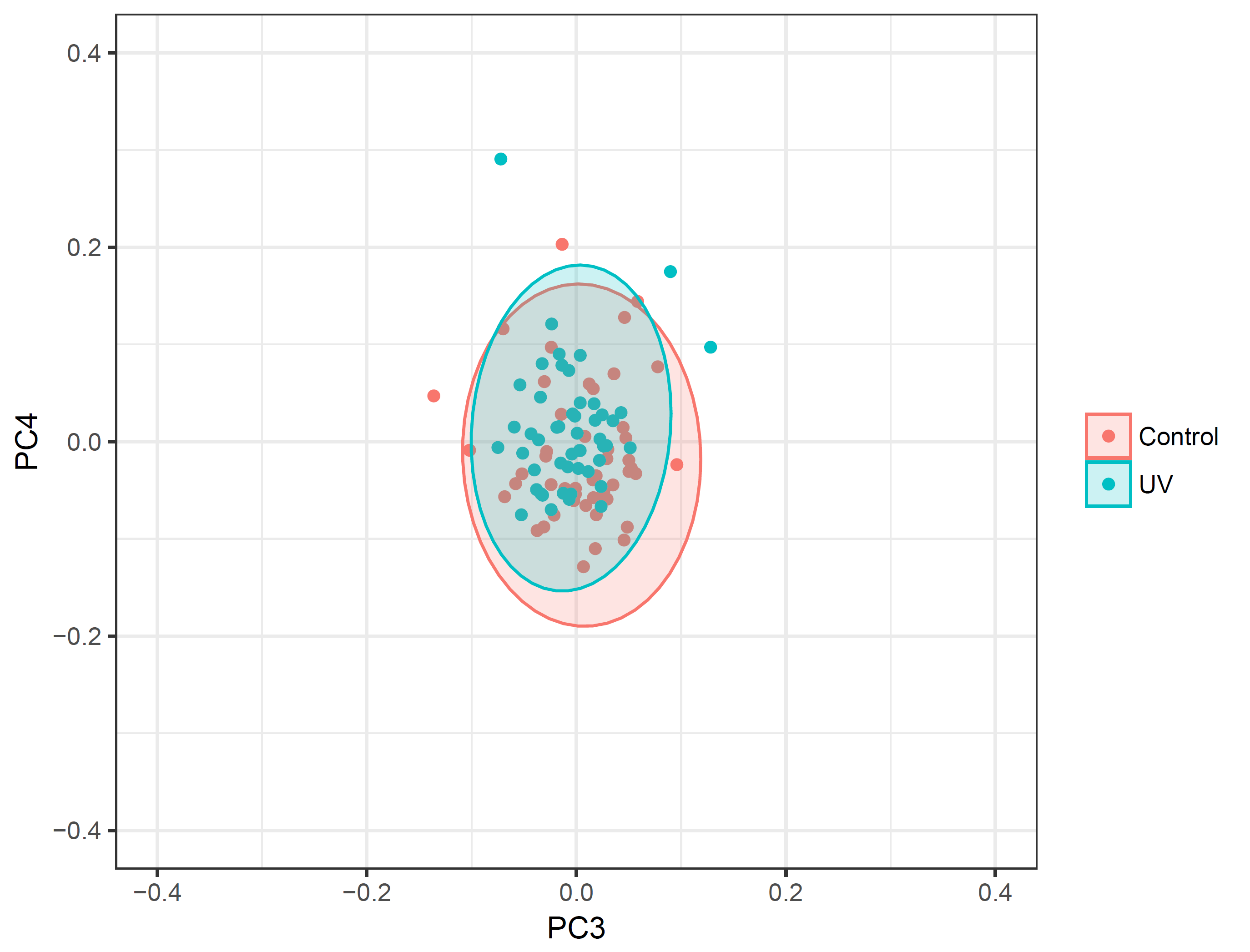

Supplement: S11 Fig — (TIF) [file pone.0207786.s011.tif]
