# Supplementary material for: Spectral features of nuclear DNA in human sperm assessed by Raman Microspectroscopy: Effects of UV-irradiation and hydration
Source: PLoS One. 2018 Nov 20;13(11):e0207786. doi: 10.1371/journal.pone.0207786 (PMC6245842; doi:10.1371/journal.pone.0207786)
Supplement: S1 Table — (DOCX) [file pone.0207786.s012.docx]

| **HTF-Buffer** | **(mM)** | **PBS** | **(mM)** | **Saline** | **(%)** |
| --- | --- | --- | --- | --- | --- |
| NaCl  KCl  MgSO_4_  KH_2_PO_4_  CaCl_2_  HEPES  Lactic acid  Sodium pyruvate  Glucose | 97.8  4.69  0.2  0.37  2.04  21  21.4  0.33  2.78 | NaCl  KCl  Na_2_HPO_4_  KH_2_PO_4_ | 137  2.7  10  1.8 | NaCl | 0.9 |
